# Anxiety and depression among Kosovar university students during the initial phase of outbreak and lockdown of COVID-19 pandemic

**DOI:** 10.1080/21642850.2021.1903327

**Published:** 2021-03-24

**Authors:** Aliriza Arënliu, Dashamir Bërxulli, Blerta Perolli - Shehu, Besnik Krasniqi, Ardian Gola, Flamur Hyseni

**Affiliations:** a Department of Psychology, Faculty of Philosophy, University of Prishtina, Prishtina, Kosovo; b Department of Early Childhood Education, Faculty of Education, University of Prishtina, Prishtina, Kosovo; c Department of Management, Faculty of Economics, University of Prishtina, Prishtina, Kosovo; d Department of Sociology, Faculty of Philosophy, University of Prishtina, Prishtina, Kosovo; e Administrative & Constitutional Department, Faculty of Law, University of Prishtina, Prishtina, Kosovo

**Keywords:** COVID-19, anxiety, depression, Kosovo

## Abstract

**Background and Purpose:** Measures taken to prevent COVID-19 infections, aside from causing disruptions in many facets of our daily life, have impeded education, including the higher education process, as never seen before on a global scale. Recent studies have indicated the potential negative impact of the COVID-19 pandemic on the mental health trajectory of university students. Anxiety and depression can seriously hamper students’ quality of life and educational achievement. This study explored anxiety and depression among university students in Kosovo during the lockdown as a result of COVID-19 pandemics. The study was implemented during the initial phase of the pandemic. This study also explored the impact of selected determinants on the reported mental health of students. **Methods:** In total, 904 university students from the largest public university in Kosovo were enrolled in a web-based cross-sectional study during the early phase of the national lockdown due to the COVID-19 pandemic. **Results:** The first and second hierarchical regression models indicated that the anxiety and depression scores of students were predicted by gender, knowing someone who was infected with COVID-19, concerns about the potential financial impact of measures taken by governments to respond to the COVID-19 pandemic and excessive information seeking about COVID-19. The third model improved significantly when the variables concerns about family members’ health, concerns about being lonely, problems with online lectures and requests for help from the university related to online lectures were added to the model. Overall, the findings indicated that situational variables associated with measures taken to respond to the COVID-19 pandemic appeared to predict anxiety and depression among students. **Conclusion:** These findings indicate that universities and public health institutions need to support students, especially the more vulnerable students, in enhancing their skills to cope with mental health problems and distress related to the pandemic and the shift to online learning.

## Introduction

Coronavirus disease 2019 (COVID-19) began as an epidemic in China in December 2019 and quickly made its way around the world in a matter of weeks. On 11 March 2020, the World Health Organization (WHO) declared COVID-19 a pandemic, due to the global outbreak of the disease (Cucinotta & Vanelli, 2020). Basic prevention measures included physical distancing and quarantine. Many countries took many other unprecedented measures to prevent the spread of the disease, including measures to remedy the effects on national economies. Measures also included closing all educational institutions, including universities, and moving to online lectures. Previous studies have indicated a relatively high prevalence of depression and anxiety among university students (Ibrahim, Kelly, Adams, & Glazebrook, 2013; Kosic, Lindholm, Järvholm, Hedman-Lagerlöf, & Axelsson, 2020; Sarokhani et al., 2013). The measures and radical changes in everyday life due to the COVID-19 pandemic potentially can worsen the mental health of students. Depression and anxiety among students can seriously negatively impact students’ quality of life and academic achievement (Bayram & Bilgel, 2008; Farrer, Gulliver, Bennett, Fassnacht, & Griffiths, 2016).

The COVID-19 pandemic is a public health emergency that can affect the mental health of individuals. Public health emergencies can cause insecurity, confusion, emotional isolation, and economical losses at the individual and community levels, work and school closures, and inadequate distribution of medical resources (Pfefferbaum & North, 2020). In many other countries, pandemic prevention measures, such as national lockdowns, working from home, and the limitation of social contact, were associated with elevated levels of stress, anxiety, depression, and negative emotions in the general population (Cooke, Eirich, Racine, & Madigan, 2020; Li, Wang, Xue, Zhao, & Zhu, 2020; Mazza et al., 2020; Nelson, Pettitt, Flannery & Allen, 2020; Okan et al., 2020; Salari et al., 2020).

The first two cases of infected persons with COVID-19 in Kosovo were identified on 13 March 2020. In September 2020, there were already more than 15,000 COVID-19 cases and more than 600 deaths due to this virus (Open Data Kosovo, n.d.). The government of Kosovo introduced various measures aimed to reduce the number of infections and deaths from COVID-19, including restricting the movement of citizens, promoting physical distancing, closing schools and universities, implementing quarantines of specific geographic areas (UNDP, 2020), and taking other measures that in general paralyzed ‘normal’ life.

The closing of universities required teaching to be continued online. In general, this was a major shift for both students and teaching staff who had limited or no experience with online teaching. The pandemic has created the largest disruption of the education system ever recorded in 190 countries, where school or university closures have impacted 94% of the student population and 99% of the population in low and lower-middle-income countries (UN, 2020). Shifting from face-to-face to online classes often is associated with a lack of availability of information technology (IT) resources and Internet connection, difficulties in rearranging conditions at home for work and study, and challenges assessing and evaluating education processes and the mental health of students (Sahu, 2020).

A longitudinal study indicated that students’ levels of stress, anxiety, and depression worsened compared to that before the pandemic (Elmer, Mepham, Stadtfeld, & Capraro, 2020). The same study reported that worries shifted to increasing concerns about health, family, friends, and their future. In an 8-week follow up study with students during the COVID-19 pandemic in Croatia, students reported higher levels of stress, anxiety, and depression during the initial phases of the pandemic, with decreased intensity of symptoms over time (Vulić-Prtorić, Bodrožić Selak, & Sturnela, 2020). Students who reported having relatives or acquaintances infected with COVID-19 were three times more likely to experience increased anxiety (Cao et al., 2020; Wang et al., 2020). Cao et al. (2020) also reported that worries over economic effects, as well as delays in academic activities, were positively associated with anxiety symptoms. Students also reported more concerns about academic performance and decreased social interaction due to situations created by the pandemic (Son, Hegde, Smith, Wang, & Sasangohar, 2020). Studies in other countries have also reportedanxiety and depression during the COVID-19 pandemic among students (Islam et al., 2020; Marelli et al., 2020; Mechili et al., 2020; Odriozola-González, Planchuelo-Gómez, Irurtia, & de Luis-García, 2020; Vulić-Prtorić et al., 2020).

A previous study with 70 students in Kosovo demonstrated that the majority of participants reported moderate levels of stress and mild psychological and somatic anxiety, which were associated with problems in time management, procrastination and an inability to live a campus life (Hyseni-Duraku & Hoxha, 2020). Further exploration of the impact measures related to pandemic prevention on the mental health and psychosocial function of students is needed to better understand the impact of emergencies that might occur in the future. The current study was conducted when a lockdown was introduced in Kosovo, during which its citizens could leave their houses for only 90 min during a specified time period of day depending on the last number on their identification cards.

The findings from this study will orient and guide universities and other institutions on the mental health of students and potential risk and protective factors identified during the lockdown period as measures to attenuate the COVID-19 pandemic.

## Methodology

### Study design

This was a cross-sectional study using data collected online using Google Forms. The questionnaire was sent to the official university email addresses of students by coauthors who were teaching online courses during the COVID-19 lockdown.

## Sample

The target populations of this study were undergraduate and graduate students of the University of Prishtina ‘Hasan Prishtina’, the largest university in Kosovo. The questionnaire was sent to a total of 1770 students out of 42,000 registered students of University of Prishtina ‘Hasan Prishtina’. The students who were invited to participate in the study were from four different faculties of the university: the Faculty of Philosophy (500), Faculty of Economics (300), Faculty of Law (200) and Faculty of Education (600). In total, 904 (51.07%) students responded by completing the questionnaire: 290 from the Faculty of Philosophy, 146 from the Faculty of Economics, 145 from the Faculty of Law and 323 from the Faculty of Education. The questionnaire was distributed during the third and fourth weeks of the pandemic when the country was in lockdown. Data were collected within a time span of 10 days, and no reminder to complete the questionnaire was sent to students. The lockdown measures included strict movement restrictions, where individuals had the right to be outside 90 min per day during specific hours of the day depending on the last digit on their ID. Each student provided consent to participate in the study and for the usage of their anonymous data for research purposes.

### Ethics statement

Ethical approval for the study was granted from the ethical board of the higher education institution where the research took place.

## Measures

The questionnaire included basic demographic information, such as age, gender, and field of study. The following additional questions were asked: ‘Did you experience isolation because you were suspected to be infected?’ (Yes/No); ‘Do you know anyone who tested positive for COVID-19?’ (No/Yes, one person/More than one person); ‘Has anyone from your family or close friends tested positive for COVID-19?’ (No/Yes, one person/More than one person); ‘Are you worried about financial problems due to the restriction measures?’ (Not worried at all/Slightly worried/Moderately worried/Somewhat worried /Very worried); ‘Do you actively search for information about the progress of the coronavirus pandemic (e.g. read news websites, use apps to be updated, listen to the radio/TV, etc.) to learn about new developments?’ (Not at all/A little/Moderately/A lot/Extremely); ‘Are you experiencing difficulties in online lectures?’ and ‘Are you experiencing difficulties in educational assignments/coursework?’ (No difficulties experienced/Little difficulties/Quite some difficulties/ A lot of difficulties). The need for help from the university in terms of online courses was assessed by a single question (No need for help/Maybe a little/Yes, I need help).

Anxiety and depression were assessed using the HADS (Hospital Anxiety Depression Scale). The measure was designed to detect and screen anxiety and depression in individuals with physical illness but has been extensively used in various populations, including nonclinical populations (Snaith, 2003). The scale has 14 items (7 items assessing anxiety and 7 items assessing depression), with scores ranging from 0 to 3 for each item. Higher scores indicate a higher severity of anxiety and depression, with a specified range of scores for normal, mild, moderate, and severe levels of anxiety and depression (Bjelland, Dahl, Haug, & Neckelmann, 2002). The measure was previously used in a study in Kosovo (Nakayama et al., 2014), with Cronbach alpha coefficients of .68 for anxiety and .72 for depression. The Cronbach alpha coefficients in the current study were .77 for anxiety and .91 for depression.

## Data analysis

Initially, the percent distribution was calculated, mean comparison was conducted, and the correlation of variables was determined. Second, hierarchical linear regression analysis was used to explore the association between demographic variables in the first step, and in the second step, direct experiences related to COVID-19, such as being isolated due to virus and having acquaintances who tested positive for COVID-19, were analyzed. The third model included two concerns related to the latest situation (loneliness and economic concerns) and reported difficulties with online courses. SPSS 24 (Statistical Program for Social Sciences) was used for data analysis.

## Results

This study explored the reported anxiety and depression of university students and their association with relevant life experiences during the COVID-19 pandemic.

The age of participants ranged from 17 to 53; the average age was 20.97 (SD = 3.4). The majority of respondents were female (769; 85.1%), and there were 135 males (14.9%). Female students reported significantly higher average scores for anxiety and depression than male students, as shown in Table 1. Only 14 (1.5%) participants experienced isolation due to having potentially been in contact with an infected person. One hundred thirty-nine (15.4%) students declared they knew someone who was infected with COVID-19, and only 33 (3.7%) were family members or close friends. Students who reported knowing someone who tested positive for COVID-19 reported significantly higher average scores for anxiety and depression than students who reported not knowing anyone. Students who reported having family members or close friends infected with COVID-19 reported significantly higher average scores for anxiety but not depression than those who did not have family members or close friends infected with COVID-19. Nearly half of the students reported that they might need help from the university for support for online courses 511 (56.7%), and 73 (8.1%) reported they definitely needed help. Students reporting a need for help from the university reported significantly higher average scores for anxiety and depression than students reporting no need for help (Table 1).

**Table 1.** Individual and Covid-19 pandemics contextual related experiences and concerns association with anxiety and depression.

Variables	Frequency	Anxiety, mean (SD)	Depression, mean (SD)
Gender			
Female	769 (85.1)	6.70 (3.97)	5.94 (3.56)
Male	135 (14.0)	4.90 (3.46)	5.22 (3.46)
		[*t* (889) = *t* = 4.937; *p* = .001*]	[*t* (885) = 2.110; *p* = .035*]
Experienced isolation doubted infection?			
No	890 (98.5)	6.42 (3.94)	5.81 (3.55)
Yes	14 (1.5)	6.71 (4.77)	7.07 (3.55)
		[*t* (889) = –.270; *p* = .826]	[*t* (885) = –.1282; *p* = .226*]
Do you personally know someone was tested positive with the COVID-19?			
No	763 (84.6)	6.23 (3.84)	5.66 (3.51)
Yes	139 (15.4)	7.54 (4.36)	6.77 (3.63)
		[*t* (889) = –3.295; *p* = .001*]	[*t* (885) = –3.347; *p* = .001*]
Has anyone from your family or close friends been tested positive with the COVID-19?			
No	871 (96.3)	6.37 (3.94)	5.79 (3.53)
Yes	33 (3.7)	8.03 (3.91)	6.88 (3.94)
		[*t* (889) = –2.380; *p* = .023*]	[*t* (885) = –1.560; *p* = .128*]
Do you need help from your university?			
1. No need for help	318 (35.3)	4.98 (3.49)	4.70 (3.28)
2. Maybe a little	511 (56.7)	7.09 (3.90)	6.26 (3.50)
3. Yes I need help	73 (8.1)	8.22 (4.23)	7.73 (3.67)
		*F*(3,890) = 38.96; *p* = .001, 1 < 2*, 1 < 3*,	*F*(3,886) = 15.564; *p* = .001, 1 < 2*, 1 < 3*, 2 < 3*

**Table 2.** Severity of anxiety and depression according to cut of scores.

	Normal	Mild	Moderate	Severe	Missing
Anxiety	560 (61.9)	202 (22.3)	98 (10.8)	33 (3.7)	11 (1.2%)
Depression	637 (70.5)	167 (18.5)	71 (8)	14 (1.5)	15 (1.7%)

The vast majority of the students 558 (61.9%) reported scores falling within normal levels for anxiety, 202 (22.4%) reported mild levels of anxiety, 98 (10.9%) reported moderate levels of anxiety, 33 (3.7%) reported severe levels of anxiety, and 11 (1.2%) participants had missing values for anxiety. For depression, 635 (70.4%) reported normal levels of depression, 167 (18.5%) reported mild levels of depression, 71 (7.9) reported moderate levels of depression, 33 (1.7%) reported severe levels of depression, and 15 (1.7%) had missing values for depression (Table 2).

Table 3 presents the means and standard deviations of the variables and their intercorrelation values. The intensity of information seeking related to the COVID-19 pandemic was significantly positively correlated with anxiety (*r* (891) = .209, *p* = .01) and with depression (*r* (887) = .153, *p* = .001). Concerns about financial problems were significantly positively correlated with anxiety (*r* (883) = .192, *p* = .01) and depression (*r* (879) = .222, *p* = .01). Concerns about being lonely due to the lockdown were positively correlated with anxiety (*r* (881) = .489, *p* = .001) and with depression (*r* (876) = .521, *p* = .01). Worrying about family members’ health was positively correlated with anxiety (*r* (884) = .376, *p* = .001) and with depression (*r* (879) = .280, *p* = .01). Reporting difficulties with online lectures was positively correlated with anxiety (*r* (883) = .296, *p* = .001) and with depression (*r* (878) = .326, *p* = .01). Similarly, reporting difficulties with course-related projects during the lockdown was positively correlated with anxiety (*r* (890) = .279, *p* = .001) and with depression (*r* (887) = .304, *p* = .01).

**Table 3.** Bivariate correlations between Covid-19 pandemics contextual related experiences and concerns association with anxiety and depression.

	*n*	*M* (SD)	1	2	3	4	5	6	7	8
1. Anxiety	891	6.43 (3.95)	1							
2. Depression	887	5.83 (3.56)	0.712**	1						
3. Seek information	902	3.38 (1.03)	0.209**	0.153**	1					
4. Concern financial problems	894	2.37 (1.29)	0.192**	0.222**	0.098**	1				
5. Concern loneliness	890	1.91 (.965)	0.489**	0.521**	0.057	0.171**	1			
6. Concern family health	893	2.59 (1.01)	0.376**	0.280**	0.134**	0.159**	0.218**	1		
7. Problems online lectures	892	2.12 (1.02)	0.296**	0.326**	−0.039	0.094**	0.238**	0.183**	1	
8. Problems course projects	899	2.31 (1.05)	0.319**	0.344**	−0.032	0.116*	0.312**	0.236**	0.753**	1

Table 4 presents the results of the hierarchical regression analysis for the anxiety scores of university students. In model 1, only gender significantly predicted anxiety scores (*r* (*β* = –.167; *p* < .01)) and contributed to approximately 3% of the variance (Δ*R*² = .03, *F* (1, 869) = 13.52; *p* < .083). In the second model, the personal experience variables related to COVID-19, including being female (*β* = –.155; *p* < .01), knowing someone who was infected (*β* = .125; *p* < .01) and seeking information related to COVID-19 (*β* = .199; *p* < .01), were entered and predicted 8% of the variance (Δ*R*² = .0, *F* (1, 869) = 13.520; *p* < .01). Last, in the third model, in addition to the personal variables of gender and age and the personal experience variables related to COVID-19, we added reported concerns about loneliness due to restriction measures, concerns about family members’ health, two items measuring reported difficulties with online courses and projects and, finally, the need for help from the university with online courses. These variables significantly increased the variance in the anxiety scores to 39.1% (Δ*R*² = .391, *F* (1, 863) = 47.480; *p* < .01), and the variables that significantly predicted anxiety in this model were being female (*β* = –.132; *p* < .01), knowing someone who was infected (*β* = .060; *p* < .05), seeking information related to COVID-19 (*β* = .149; *p* < .01), being concerned about loneliness (*β* = .362; *p* < .01), being concerned about family members’ health (*β* = .204; *p* < .01), having problems with online courses (*β* = .104; *p* < .05) and reporting the need for help from the university (*β* = .129; *p* < .01).

**Table 4.** Summary of hierarchical regression analysis for variables predicting anxiety among university students during COVID-19 lockdown.

	Model 1			Model 2			Model 3		
	*B*	St. err	Beta	*B*	St. err	Beta	B	St. err	Beta
Gender (1 = female)	−1.836	0.369	−1.67**	−1.705	0.360	−0.155**	−1.453	0.295	−0.132**
Age	−0.044	0.042	−0.035	−0.036	0.041	−0.029	−0.001	0.842	−0.001
Isolation risk infection				0.663	1.028	0.021	−0.151	0.842	−0.005
Know someone infected				0.910	0.264	0.125**	0.434	0.217	0.060*
Family or friend infected				−0.101	0.551	−0.007	0.341	0.451	0.022
Seek information				0.760	0.125	0.199**	0.570	0.103	0.149**
Concern loneliness							1.485	0.117	0.362**
Concern family health							0.802	0.111	0.204**
Problems online lectures							0.402	0.159	0.104*
Problems course projects							0.069	0.157	0.018
Needs assistance from university							0.854	0.193	0.129**
*R*^2^		0.030			0.080			0.391	
*F* for change in *R*^2^		13.52**			13.520**			47.48**	

Table 5 presents the results of the hierarchical regression analysis for the depression scores of university students for the same set of variables as in the previous model for anxiety. In model 1, being female significantly predicted anxiety scores (*r* (*β* = –.075; *p* < .05)) and contributed very little to the variance, with a nonsignificant result for the overall model (Δ*R*² = .03, *F* (1, 864) = 2.449; *p* < .087). In the second model, the personal experience variables related to COVID-19 were entered, including being female (*β* = –.066; *p* < .05) knowing someone infected with COVID-19 (*β* = .124; *p* < .01) and seeking information related to COVID-19 (*β* = .148; *p* < .01), which predicted approximately 4% of the variance (Δ*R*² = .037, *F* (1, 864) = 6.445; *p* < .01). Last, in the third model, in addition to the personal variables of gender and age and the personal experience variables related to COVID-19, we added reported concerns about loneliness due to restriction measures, concerns about family members’ health, two items measuring reported difficulties with online courses and projects and the need for help from the university related to online courses. These variables significantly increased the variance in depression scores to 36.8% (Δ*R*² = .368, *F* (1, 864) = 42.932; *p* < .01), and the variables that significantly predicted depression in this model were knowing someone infected with COVID-19 (*β* = .058; *p* < .05), seeking information related to COVID-19 (*β* = .115; *p* < .01), being concerned about the financial impact of measures related to the pandemic (*β* = .095; *p* < 0.01), being concerned about the health of family members (*β* = .106; *p* < .01), being concerned about loneliness (*β* = .413; *p* < .01), having problems with online courses (*β* = .135; *p* < .01) and reporting the need for help from the university (*β* = .075; *p* < .05).

**Table 5.** Summary of hierarchical regression analysis for variables predicting depression among university students during COVID-19 lockdown.

	Model 1			Model 2			Model 3		
Gender	−0.743	0.338	−0.075*	−0.656	0.333	−0.066*	−0.463	0.272	−0.047
Age	−0.002	0.038	−0.002	0.004	0.038	−0.004	0.037	0.031	0.033
Isolation risk infection				1.442	0.979	0.049	0.399	0.800	0.014
Know someone infected				0.815	0.246	0.124**	0.381	0.201	0.058*
Family or friend infected				−0.496	0.509	−0.036	−0.110	0.414	−0.008
Seek information				0.511	0.115	0.148**	0.395	0.095	0.115**
Concern loneliness							0.264	0.078	0.095**
Concern family health							0.374	0.101	0.106**
Problems online lectures							0.471	0.146	0.135**
Problems course projects							0.179	0.144	0.053
Needs assistance from university							0.447	0.177	0.075*
*R*^2^		0.003			0.037			0.368	
*F* for change in *R*^2^		2.449			8.401**			42.932**	

## Discussion

Mental health distress is common during public health emergencies (Pain & Lanius, 2020). The current pandemic is considered one of the most wide-reaching global challenges. As there are uncertainties about the disease and its treatment, including the lack of a vaccine for the virus, it appears that a lack of clarity of societal responses will continue for some time (Atchison et al., 2020). This lack of clarity and unpredictability might adversely affect the mental health of individuals.

The primary aim of this study was to evaluate the mental health of university students in Kosovo during the early phase of the COVID-19 pandemic and to explore the impact of certain factors associated with students’ mental health. This study was conducted during the third and fourth weeks of the national lockdown due to COVID-19 when online lectures were ongoing. During this period, many unknowns related to the epidemic were present, including the future of education and career opportunities. Depression and anxiety levels in this study were lower than those reported in Bangladesh, where two-thirds of students scored above the normal cut off scores for anxiety (Islam, Barna, S.Raihan, Khan, & Hossain, 2020). However, the findings on the severity of anxiety were similar to findings from studies exploring the mental health of students during pandemics, such as for anxiety in China (Cao et al., 2020), Italy (Marelli et al., 2020) Croatia (Vulić-Prtorić et al., 2020), Spain (Odriozola-González et al., 2020) and Albania (Mechili et al., 2020). The findings from the current study indicate that situational variables related to the COVID-19 pandemic, such as seeking excessive information related to COVID-19, knowing someone who tested positive for COVID-19, expressing concerns about family members’ health, reporting concerns about being lonely and the economic consequences of COVID-19-related measures, and reporting problems with online courses, significantly predicted anxiety and depression levels among students in Kosovo. Hierarchical regression analysis indicated an increase in the predictive power when the last four mentioned variables were added to the model, including the need for help from the university regarding online courses. The findings of this study are in line with those of other studies in which economic concern was reported to be associated with psychological distress among students (Cao et al., 2020) and the general population (Xiong et al., 2020). Similar findings have been reported for extreme information seeking and its impact on anxiety (Garfin, Silver, & Holman, 2020; Holman, Thompson, Garfin, & Silver, 2020; Liu & Liu, 2020) and the experience of problems with online courses (Idowu, Akinola Olawuyi, & Nwadioke, 2020; Sahu, 2020).

The findings indicate that universities should consider providing psychosocial support to their most vulnerable students, either by providing online psycho-educational information or intervening on mental health problems and coping strategies, which have been shown to be efficient and acceptable in other studies and similar contexts (El Morr, Ritvo, Ahmad, & Moineddin, 2020; Harrer et al., 2019; Naslund et al., 2017; Wasil, Taylor, Franzen, Steinberg, & DeRubeis, 2020). Helping students enhance their coping skills in response to mental health distress can further help students to lower the impact of pandemics and similar situations. Special attention should be provided to students who are more vulnerable in terms of social isolation, risk of infection and loss of family members as a result of COVID-19. As it appears that online courses will be continued into the next semester, universities should consider additional online activities to improve public health messaging, including regarding the mental health of students. Furthermore, in times of pandemics, faculty and staff should provide virtual office hours to students to help them identify alternative internships that enable them to work from home (Zhai & Du, 2020).

The current study has several limitations, including its cross-sectional design and a lack of baseline data to compare variables in focus before the pandemic. Following up with these students longitudinally could provide more information on changes in their mental health and how these situational variables might have differential impacts on outcome variables, such as COVID-19 related measures being eased during summer 2020. Also, the current study included students only from certain faculties where authors were teaching. The current study did not consider previous mental health problems, experiences or other psychosocial conditions, which might have been present and affected students’ mental health. Furthermore, another limitation of the current study is on the factor structure of dependent variables measure used which recommend that HADS rather measures psychological distress rather separately anxiety and depression (Cosco, Doyle, Ward, & McGee, 2012; Norton, Cosco, Doyle, Done, & Sacker, 2013) therefore findings from the current study could be considered as an indication also of psychological distress rather specifically anxiety and depression.
